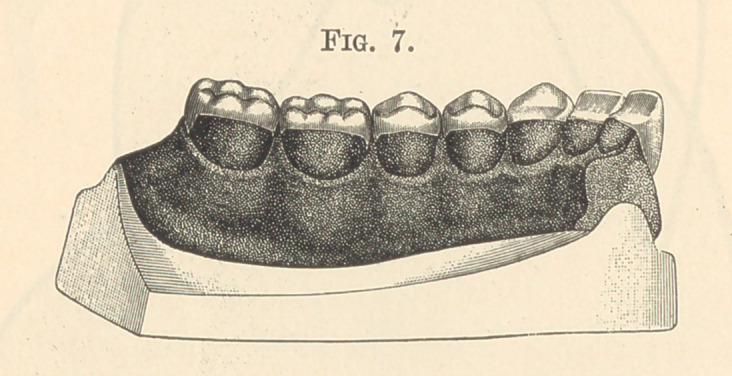# A Method for Reproducing the Natural Contour of Artificial Teeth on the Lingual and Palatal Surfaces of Artificial Dentures

**Published:** 1904-08

**Authors:** William Middleton Fine

**Affiliations:** Philadelphia


### International Dental Journal.

Vol. XXV. August, 1904. No. 8.

### Original Communications.[1]

### A METHOD FOR REPRODUCING THE NATURAL CONTOUR OF ARTIFICIAL TEETH ON THE LINGUAL AND PALATAL SURFACES OF ARTIFICIAL DENTURES.[2]

BY WILLIAM MIDDLETON FINE, D.D.S., PHILADELPHIA.

Mr. President and Members of the Academy of Stomatology,—In describing this method for making and finishing dentures, particularly vulcanite work, I shall refrain from going into detail regarding the best methods for taking impressions, running models, etc., but I will state at once that a good plaster-of-Paris impression is positively essential for the attainment of a good result.

At the thirty-third annual meeting of the New Jersey State Dental Society, held at Asbury Park, N. J., July, 1903, I had the pleasure of meeting Dr. George H. Wilson, Professor of Prosthesis and Metallurgy, Department of Dentistry of the Western Reserve University, and while watching one of his demonstrations, the idea

---

[1] The editor and publishers are not responsible for the views of authors of papers published in this department, nor for any claim to novelty, or otherwise, that may be made by them. No papers will be received for this department that have appeared in any other journal published in the country.

[2] Read before the Academy of Stomatology, Philadelphia, January 26, 1904.

of carving the wax to obtain the natural contour of the teeth on the lingual and palatal surfaces came to me. Therefore this method is not entirely original.

Dr. George B. Snow, of Buffalo, N. Y., published an article in the *Dental Advertiser,* April, 1899, relating to the Lingual Conformation of Dental Plates; but Dr. Snow confines his ideas to the rugæ and the thickness of the plate. The late Dr. Charles J. Essig also made reference to a method for carving the lingual and palatal surfaces of artificial dentures. Again let me state that I do not claim priority nor originality in method of applying the rugæ, tinning the models, etc., but from what I have been able to learn from men of prominence in the dental profession, and from examination of our literature, I feel justified in saying that the method of carving the wax to obtain the natural contour of the teeth on the lingual and palatal surfaces, to which I have the pleasure of directing your attention, has not heretofore been published or generally used.

First, we take a good plaster-of-Paris impression, and if a vacuum chamber is used, one should be carved in the impression. The next step is the model, from that the wax bite, then the articulation and the making of the base plate and the trying in of the piece. Every dentist is familiar with this phase of the work. The teeth are set up in the usual way in wax. The gums are carved and the whole denture made to reproduce the lost teeth and supporting tissues. After this operation is carried out in detail, the case is tried in the mouth, when, if found to be perfectly satisfactory in every way, the patient is dismissed and the case taken to the laboratory for finishing. Here is where we commune with nature; we strive to reproduce the beautiful and natural, the teeth and gums, the rugæ and all the peculiarities of the roof of the mouth. We try to be artistic and practical at the same time, though it is hardly possible that the dentist will ever be able to make an appliance so near to nature's models that one cannot detect the difference.

In carving the lingual and palatal surfaces to represent the natural teeth, I have a small spatula that I use for this particular work. It is made from a broken plug finishing file No. 101, S. S. White, and shaped like the diagram. (Fig. 1.)

The end A is the most used in contouring the wax, as I will describe. Take the articulator in your left hand and open it so that the upper denture is in front of you and at the top; start at the last tooth on the left side and proceed to carve the wax, using

FIG. 1.

A

FIG. 2.

point A of the wax spatula, carved downward, using the palatal surface of the porcelain tooth as your guide, and shape up the wax to represent the natural tooth as it stands in the natural gum. (Fig. 2.)

After you have formed the palatal surface of the second molar, proceed to the first, and so on around the arch of the denture until you have completed the work. Then apply the rugæ. The method of technique taught by Dr. A. Dewitt Gritman to the Freshman classes at the University of Pennsylvania is employed. To do this one must have several pure tin or zinc dies; tin is best. Take an impression of your own mouth to start with, or, better still, if the patient has well-defined rugæ, take an impression and make the die; after you have the die, take a piece of No. 40 tin-foil and burnish it over the rugæ on the die, using the rounded end of a lead-pencil eraser to press the foil into contact with the die. If a metal burnisher is used, it will be likely to tear holes in the tin-foil, but a soft eraser or piece of rubber will give a good impression and will not do any damage. A piece of tin-foil should be used large enough to cover the entire palatal surface of the wax denture, and have a free margin of tin-foil one-quarter inch in addition. After the burnishing is completed, remove carefully and fill the impressions in the tin-foil (the side that comes next to the die) with wax, by melting it on a spatula and dropping it on the tin-foil; then heat the spatula quite hot and smooth it down. By this means we have a smooth surface that is brought into contact with the palatal surface of the wax base-plate. After this is done, press the piece, wax side down, into the wax base-plate and finish around the teeth by burnishing very carefully, but do not burnish the edges of the tin-foil down flat, allow it to stand up at right angles so that when the case is invested the free edges will be embedded in the plaster and hold the tin-foil in place. The result is a tin-foil covering to the palatal surface of the wax denture, and after the tin-foil has been applied to the gums, to which it is burnished on in the same way, the case is ready to flask. After the wax has been boiled out, cover the model with very thin tin-foil, No. 4 or 6. We have now a tin-faced matrix in which to pack the rubber. The case is then packed with rubber in the usual way, placing the pink rubber for the gums in around the teeth first, after this is completed pack in the red rubber. Close the flask and vulcanize.

Now, a few words regarding flasking, finishing, etc., etc. Flask the cases in the usual way; any style flask may be used; separate the flask, remove wax, pack in rubber, and place a piece of the waxed cloth (that which comes over the rubber as we purchase it from the dental depots) between the two sections of the flask so that you may separate them and ascertain if you have enough rubber; do not have too much and do not cut too many vents for the surplus material to flow out; if you do, the rubber will not be under the same steady pressure when vulcanizing that it should be. Do not close the flask with too much pressure at first, do this gradually. After the flask is closed, place it in the vulcanizer with cold water, fasten the lid down tight, and proceed to vulcanize. Allow the vulcanizer to run for about ten minutes and then open the safety valve and let the air out. Then take from one-half to three-quarters of an hour to run the vulcanizer up to 320°, and then vulcanize at that point for one hour and a half. This will give you a good substantial plate and one that will take a very high polish. In finishing the cases, it is necessary to scrape but very little if the case has been properly waxed up and tinned. If necessary to scrape, use a very sharp pointed scraper and scrape around the teeth and between them first, the rest of the plate in the usual way. Polish with pumice and felt cone, pumice and stiff brush-wheel, chalk and stiff brush-wheel, chalk and soft brush-wheel, and last a very soft brush-wheel and rouge. If a greater polish is desired, use a chamois wheel and rouge. A still greater polish may be obtained with dry plaster of Paris and oil, using the finger-tip to polish with. Clean the rouge away from the plates with a soft laboratory brush and castile soap.

By this method dentures can be made much thinner and present a more artistic appearance, and they are more natural. They are sure to please those who are unfortunate enough to be compelled to use an artificial appliance. This method may be used in partial cases, and is by no means confined to a full case. I use it in partial cases, both vulcanite and gold with vulcanite attachments. Even in a case of three teeth the patient will tell you how much lighter and thinner the plate feels. They can speak more distinctly and it is easier for them to masticate their food.

Instead of finishing the denture up smooth to the porcelain teeth along the masticating surfaces, as shown in Fig. 3, the case is given a more natural appearance along the gum line on the palatal surface, as shown in Fig. 4. In Fig. 3 is presented the older method of waxing up the case, but in Fig. 4 is given the newer method of carving to represent the natural tooth. One can easily appreciate the "feel" of the denture to the patient and how much lighter the denture is in weight.

Fig. 3.

Fig. 4.

Fig. 5.

Fig. 5 shows section of a full case finished and ready for the mouth. Sometimes I carve the wax in such a way as to allow of the free passage of floss-silk between the teeth to the gum line. The saliva fills the spaces between the teeth on the denture in the same way that it does in a normal mouth. This doubtless aids in the articulation of words, also in the mastication of food by allowing the saliva to flow down the outside of the plate, over and between the teeth, as it does normally and mixes more thoroughly with the food. The rugæ also aids in the articulation of words and mastication of food. When the palatal surface of the

Fig. 6.

denture is smooth, the tongue has but little power to hold a morsel of food upon it, while with the rugæ the food is easily managed. The importance of the rugæ has been set forth by Dr. Burchard, Dr. Harrison Allen, Dr. Charles J. Essig, Dr. George B. Snow, and others. Some professional men object to the spaces between the teeth on artificial dentures, claiming they are too difficult to keep clean. Is it not the proper thing to clean one's own teeth with floss-silk? Why not a denture? Those who wear artificial appliances should take as good care of them as those who have all the natural teeth in place, using a tooth-brush, floss-silk, tooth-powder, and antiseptic washes.

Articulate speech consists of a modification of the voice by means of the lips, tongue, teeth, palatal arch, and rugæ, and various modifications of the oral cavity and its contents. The true sounds come from the vocal cords. The dome of the oral cavity assists in the modification, and I believe if the spaces are placed between the teeth on dentures, to correspond to the natural teeth, there should

Fig. 7.

be nearly the same modification of the voice. Of course, a denture, no matter what kind or style, changes the voice slightly. We get nearer to nature by reproducing the rugæ and each tooth accurately.

The peculiar metallic hissing sounds produced by an attempt to pronounce the letter "s," especially in such words as "susceptibilities," involving a multiplication of "s" sounds when a smooth surface is given to the denture, is entirely obviated by the use of the rugæ. The sound is broken when it comes in contact with the rugæ and is softened. Or, for example, in singing or talking in a vacant room, the voice has a certain tone. Now, if the room be changed by building off the corners with boards or curtains, the tone of the voice is also changed. This same principle holds good in prosthetic dentistry. Another example: A bridge from the first bicuspid to the third molar, the space between filled with dummies; a singer will tell you that such a piece will change the voice, sometimes to such an extent that it is almost impossible to reach certain notes. It is evident that we must try to reproduce the natural mouth as nearly as possible.

The lower denture should be made in the same way as the upper denture, with the exception of the rugæ, etc. There is a slight depression below the necks of the teeth on the lingual aspect of the lower jaw in the normal mouth. This is reproduced in the lower denture, as is shown diagrammatically in Fig. 6.

Fig. 7 shows a section of a full lower case ready for the mouth.

## Figures and Tables

**Fig. 1. f1:**
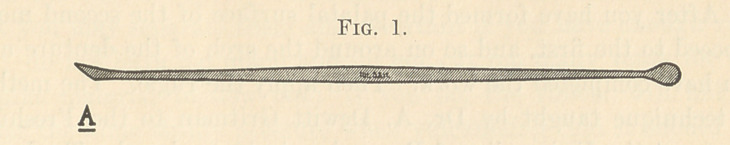


**Fig. 2. f2:**
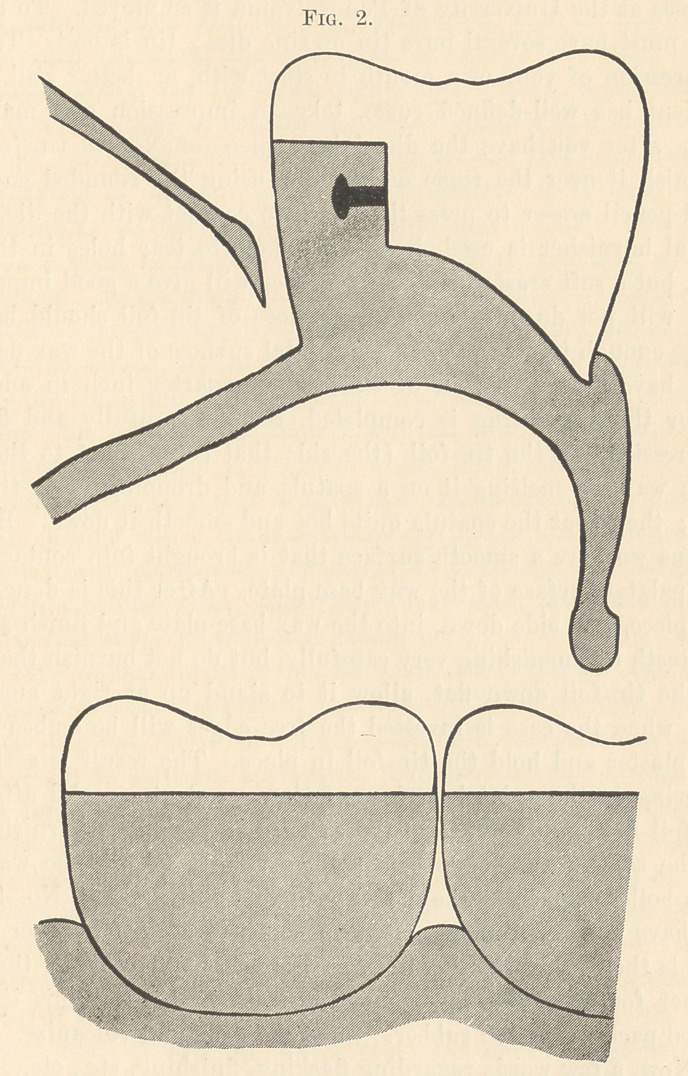


**Fig. 3. f3:**
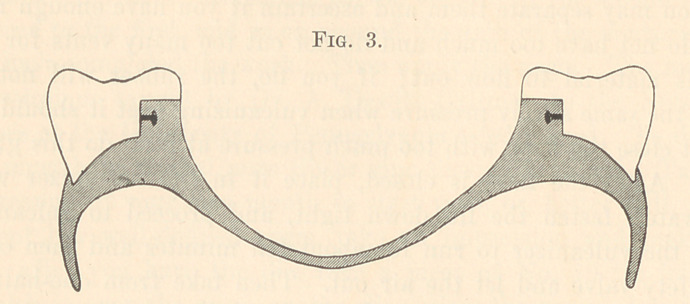


**Fig. 4. f4:**
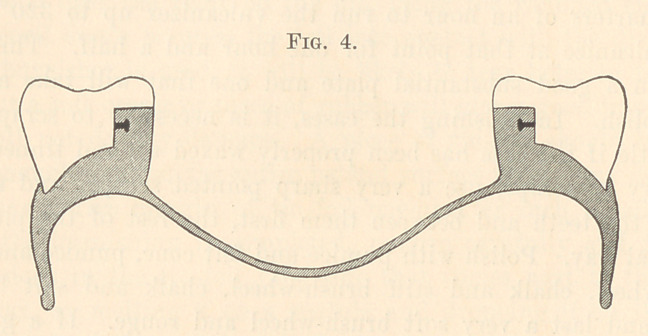


**Fig. 5. f5:**
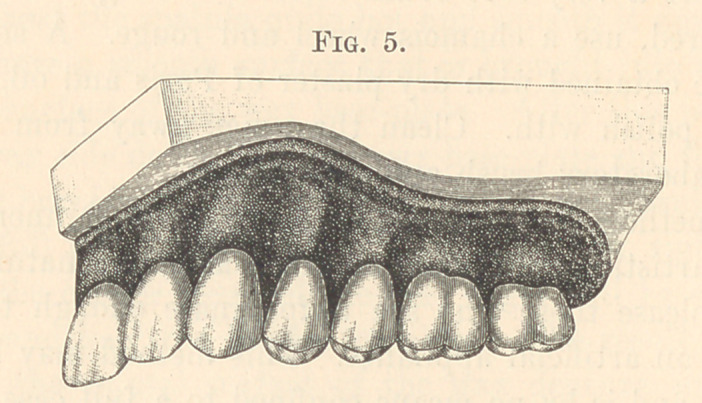


**Fig. 6. f6:**
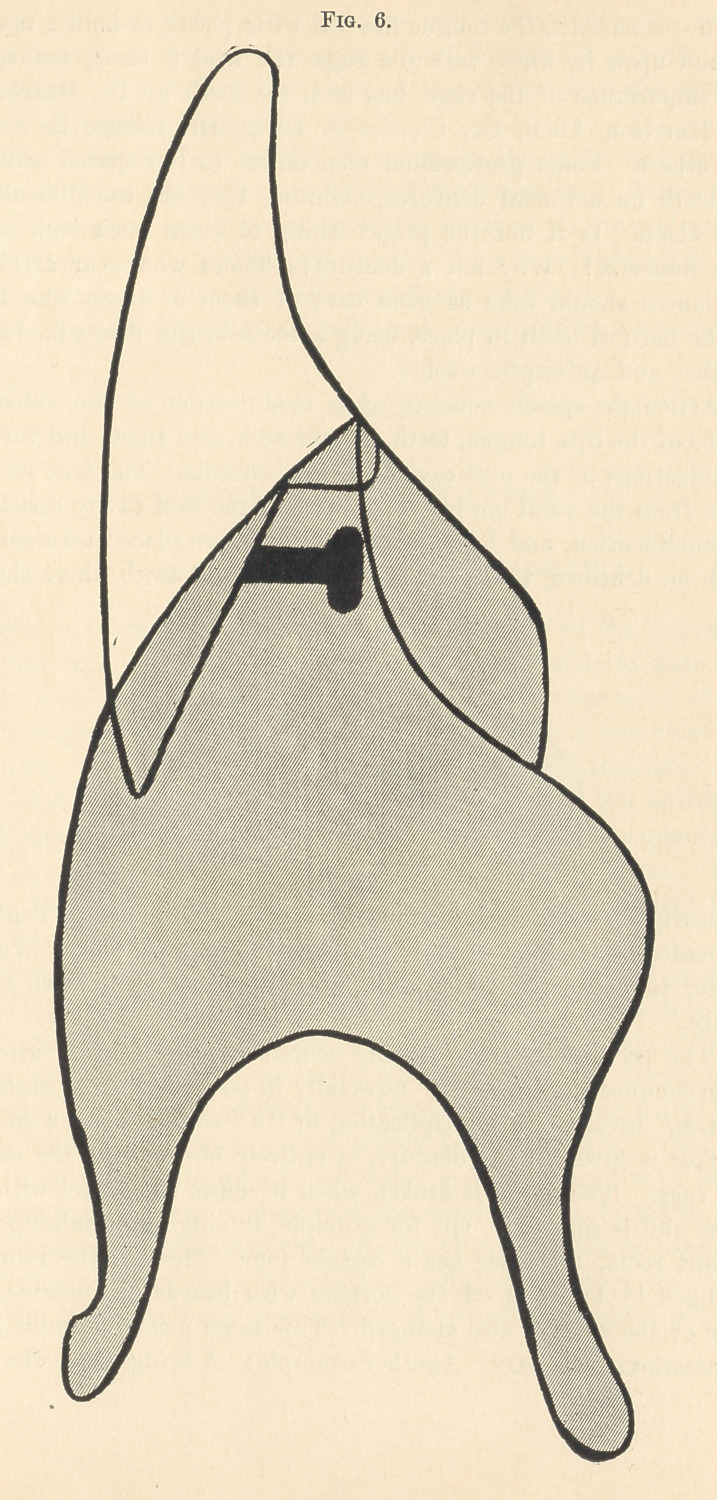


**Fig. 7. f7:**